# Histology of the Nerves: A Contribution of the Latest Researches*Translated, abridged and arranged from “Leçons sur l’Histologie du Système Nerveux,” par L. Ranvier, Professeur de l’Anatomie Générale au Collége de France. Paris. 1878. This work is beautifully illustrated with chromo-lithographs and wood-cuts, which, unfortunately, it is not practicable to reproduce here.

**Published:** 1882-11

**Authors:** Frank Dudley Beane

**Affiliations:** New York City


### BUFFALO

### Medical and Surgical Journal.

Vol. XXII. NOVEMBER, 1882. No. 4.

### Original Communications.

### Histology of the Nerves: a Contribution of the Latest Researches.*

By Frank Dudley Beane, A. M., M. D., of New York City.

II.

Having carefully examined, in the previous paper, the minute structure of nerves and nerve-tubes, we are in a position to inquire: How do nerves terminate at the periphery?

I shall enclose, within brackets, such material as has been extracted from "Leçons de Physiologie Générale et Comparée du Systéme Nerveux," par A. Vulpian, Professeur á la Faculté de Médecine de Paris, etc., etc., thus avoiding all confusion as to the balance of this letter-press, which has been derived from Ranvier's work.

[The ultimate peripheral terminations of nerves are found to consist of five varieties:

1. Subcutaneous networks.
2. Tactile corpuscles.

* Translated, abridged and arranged from "Leçons sur l'Histologie du Système Nerveux," par L. Ranvier, Professeur de l'Anatomie Générale au Collége de France. Paris. 1878. This work is beautifully illustrated with chromo-lithographs and wood-cuts, which, unfortunately, it is not practicable to reproduce here.

3. Pacinian corpuscles.
4. Corpuscles of Krause.
5. Terminal arborizations (Ranvier).

*a. Subcutaneous Networks.*—These are found within and beneath the skin and mucous membranes and other organs, endowed with sensibility. They appear as intra- or subcutaneous delicate networks of nerve axes-cylinder. In the frog there are larger or smaller nuclei in contiguous relation with the nerve-tubes of these networks, as I (Vulpian) myself have seen.

*b. Tactile Corpuscles.*—Meissner and Wagner first demonstrated this mode of termination.

These corpuscles are chiefly found in the skin of the palms and dorsum of the hands and feet, the pulps of the fingers and toes, of the glans penis, clitoris, the lips, and fungiform papillæ of the tongue. A delicate section, perpendicular to the surface of the skin in these regions, hardened in acetic acid, shows, that while some papillæ are simply vascular, others enclose a small ellipsoidal organ, with its long axis parallel to that of the papilla.

This is the *tactile*, Meissner's or Wagner's corpuscle.

Meissner and Wagner look upon them as small sacs, containing homogeneous, finely-granular, semi-fluid matter. They have a membranous external layer, showing transverse and parallel striæ. Sometimes the corpuscle receives only a single nerve-tube, sometimes two or more. The tubes preserve their double contour as far as the border of the corpuscle. They enter its interior—whether *always* is not decided—and are in contact with the internal face of its wall, taking a spiral course therein, and giving it the appearance of transverse striation (Meissner). That this striation takes its origin from the spiral course of the nerve-tube is proven by cases of atrophy of the supply nerve-tube, wherein the transverse striæ are replaced by a series of very fine oil-globules. It is probable the primitive nerve-tube (or tubes) loses its medullary and cellular sheaths at the border of the corpuscle, and that only the axis-cylinder pierces the corpuscle.

*c. Pacinian Corpuscles.*—Also called "corpuscles of Vater," after him who first described them, are found in the subcutaneous cellular tissue, generally, especially along the track of the collateral nerves of the fingers, of the sympathetic nerve-fibres, mesentery, plantar surface of the feet, dorsum of the hands and feet, fore-arm, neck, in relation with the pudic, intercostal, suborbital nerves, nerves of bones, articulations, arm and breast. They consist of a sac analogous to that of a tactile corpuscle, only it is larger and longer and is invested by several concentric layers of cellular tissue. At the centre of its pedicle a nerve-tube is seen to run as far as the wall of the sac, the latter being continuous with the nerve-tube's Schwann's sheath.

The sac's contents are soft and slightly granular, in the midst of which the nerve-tube is found. The latter consists simply of an axis-cylinder, takes usually a rectilinear, sometimes a tortuous course within the corpuscle; at times it bifurcates at a greater or less distance from its entrance.

The single axis-cylinder (or its bifurcations) often terminates as a button-like enlargement. Jacubowtsch claims they end at the summit of the corpuscle (in the cat's mesentery) in the nucleolus of a cell, several of which exist within a Pacinian corpuscle. The different investing layers of connective-tissue are separated from each other by spaces filled with a more or less fluid, transparent substance.

*d. Corpuscles of Krause* are the ultimate points of termination of nerve-tubes in mucous membranes. In structure they are simply modified tactile corpuscles. The penetration of two nerve-tubes into the interior (usually but one enters), greater fluidity of the sac contents (in certain cases), greater branching of the penetrating axis-cylinder, the ultimate termination of the axis-cylinder, or its branches, in a blunt extremity (which may sometimes show an enlargement), constitute the differences between these and the tactile corpuscles. They are found in the conjunctiva, mucous membrane of the lips, tongue (at the base of the filiform and fungoid papillæ), velum palati, glans penis and clitoris.

*e. Terminal Arborizations* (*Ranvier*) are only found in muscles. M. Rouget,* Professor of Physiology at Montpelier, France, furnished most important data upon this subject. He claimed that nerve-fibres branched a certain number of times before reaching muscles. Each branch comes in contact with the sarcolemma, wherewith the sheath of Schwann becomes confounded; the medulla disappears; the axis-cylinder penetrates the interior of the sarcolemma and becomes expanded upon the surface of the muscular substance as a granular mass, ordinarily slightly prominent, forming more or less raised plates, called by M. Rouget, *terminal motor-nerve plates.* At this point there is an accumulation of cells from the sheath of Schwann.]

M. Ranvier, by new and delicate methods, shows:

1. That it is the sheath of Henle, not that of Schwann, which becomes continuous with the sarcolemma.

2. That beneath the sarcolemma, and upon the muscular tissue itself, lies a granular substance; this latter contains special *cells*, called *fundamental*, which are very large, clear, oval, have a double contour, and very large nucleoli.

3. That this granular substance, which should be called the *nervous eminence*, is pierced by the terminal nerve-filament, which consists merely of an axis-cylinder and (presumably) Schwann's sheath, having lost its medulla at the interannular constriction previous to piercing the sarcolemma, and its sheath of Henle being confounded with the latter.

4. That this axis-cylinder *within* the nervous eminence forms itself into *arborescent branches.*

5. That these neither form loops nor anastomose with branches from other nerve-tubes. Each nerve-tube branch is free and independent of the other.

6. That these arborescent branches, *by their limbs or terminal points, pierce the granular substance surrounding them, and come into immediate contact with the muscular fibrils.*

* "Note sur la terminaison des nerfs moteurs dans les muscles chez les reptiles, les oiseaux et les mammifères." Compt. rend. de l'Acad. des Sciences; 28 Sept., 1862, t. lv, p. 548.

7. That the nervous eminences contain, besides the fundamental, two other kinds of nuclei; one kind, small and of irregular contour, situate upon the internal face of the investing membrane of the eminence, being similar to those of Henle's sheath, are called *vaginal nuclei;* the other kind, situate upon the branches of the terminal arborizations, of irregular outline, smaller than the fundamental, are called *arborization nuclei.*

8. That this terminal arborization is the form of ultimate termination of nerves in the muscles of reptiles (lizards) and mammals (rabbits). In the frog, neither granular substance nor fundamental nuclei exists; the termination occurs by short and straight stems (filaments), furnished with their respective nuclei.

Thus, M. Ranvier has penetrated further than M. Rouget, amplified the labors of Krause,* corrected the descriptions of Kühne† and Gerlach,‡ and has given us the latest and most minute investigations in this field.

In order to furnish the readers of the JOURNAL with all the data (aside from historical and technical details) contained in M. Ranvier's work, as far as he has published it (two volumes), with the exception of a description of the histology of "the electric organ of the torpedo-fish" (comprising some 132 pp. of the printed work) and of the structure of striated muscular tissue (28 pp.), we will next turn to the subject of nerve degeneration and regeneration.

*Degeneration of Nerves.*—The loss of excitability after nerve-section is complete in the rabbit, guinea-pig and rat, at the end of forty-eight hours; in the pigeon, at the end of seventy-two hours; in the dog, at the end of four days, and in the frog (in Winter) not till even the *thirtieth* day (Brown-Séquard).§

* "Ueber die Endigung der Muskelnerven;" *Archiv. f. rat. Medicin*, 1863, xviii, 136.

† "Ueber die Endigung der Nerven in den Nervenhügeln der Muskeln;" *Virch. Archiv.*, 1864, xxx, 187.

‡ "Ueber das Verhältniss der nervösen und contractilen Substanz des quergestreiften Muskels;" *Archiv. f. micr. Anat.*, 1876, xiii, 399.

§ "Recherches sur l'Irritabilité Musculaire;" *Bull. de la Soc. Philomath.*, 1847, pp. 74 et 83, and *Jour. de l'Anat. et de la Phys.*, t. ii, 75.

The younger and more vigorous the animal, the sooner the abolition of this excitability occurs; it is more rapid in warm- than in cold-blooded animals. Thus, Longet was wrong in declaring that "constantly its [the nerve's] excitability is wholly extinct *after the fourth day.*"

*At the End of Twenty-four Hours.*—If a segment of the peripheral end of a rabbit's nerve be examined (in water) twenty-four hours after section, the interannular nuclei are seen to be slightly enlarged, the nucleoli being well marked. The protoplasm which surrounds them is more abundant, being continuous with that which lines Schwann's membrane, which, in turn, has become more apparent throughout the length of the interannular segment. This protoplasmic layer has likewise become enlarged at points corresponding to the incisures of Schmidt, depressing the myelinic cylindro-cones so that the contour of the myelin of each segment is sinuous, which may be traced as a black festoon within the sheath of Schwann.

At the interannular constrictions the myelin is separated a certain distance from the sheath of Schwann which forms the terminal enlargement of the interannular constriction. The axis-cylinder may be indistinctly traced as the clearer central portion of the interior of the tube.

*At the End of Fifty Hours.*—A segment removed from a nerve at the end of this time after section shows: The nerve has lost its watered-silk appearance. In the rabbit, we know it has lost its physiological excitability. The interannular nuclei are much enlarged, contain larger and better-marked nucleoli, and are no longer applied to the internal surface of the sheath Schwann. The nucleoli are regularly spherical, generally, sometimes presenting certain irregularities to be described when speaking of the histological appearances at a later period after section.

This protoplasm has a very granular appearance, more so than in the normal condition. It also encloses fatty granulations (colored yellowish-brown by the osmic acid), and sometimes, not at all constantly, small drops of myelin (colored bluish-gray by the same reagent).

What change has taken place in the axis-cylinder? In the rabbit—the animal used in all these observations—we are able to recognize, even at *forty* hours after nerve-section, that the interannular nuclei are prominently projecting within the nerve-tubes, and that their surrounding protoplasm fills the rest of the calibre of the tube. Consequently the axis-cylinder is divided at those points. Not only has the protoplasm crowded or absorbed the myelin, but it has attacked the axis-cylinder and divided it. Some hours later, at all events at the end of fifty hours, we can observe that this protoplasm has reached and divided the axis-cylinder at points corresponding to the incisures of Schmidt.

*The Axis-cylinder Finally Disappears.*—If a peripheral segment of a nerve, which has been divided five days before, be prepared and mounted in a particular manner, microscopical examination would show that certain of the nerve-tubes are deprived of their axes-cylinder. In this transverse section, by elevating or depressing the objective, we are able to distinguish the axis-cylinder at the surface in some tubes, whilst in others it is seen to be deeply situated. That this appearance is due to the fragmentation of the axis-cylinder may be shown by another preparation. Within the nerve-tubes, the contour of which is slightly undulated, which are rose-colored, appear variable-size segments of the axis-cylinder, which are tinted red. These segments are either rectilinear or somewhat looped. The myelinic fragments are not distinguishable in this preparation. In a new preparation we see: The detached interannular nuclei, spherical in shape, colored red, and containing a well-marked nucleolus; the rose-tinted granular protoplasm; the yellow-colored, irregular fragments of myelin; and, within the latter, rose-colored segments of the axis-cylinder. We perceive that the axis-cylinder fragments are *completely* enveloped by the segments of myelin, even their extremities. This is due to the elasticity of the axis-cylinder, whereby its extremities are within its envelope, and the sides of the latter coalesce to form the rounded ends of myelin which are seen. Laying aside the consideration of the changes occurring in the axis-cylinder for awhile, let us investigate the alterations which are taking place, *pari passu*, in the other structures of the tubes.

*The fourth day after section*, in the rabbit, rat, guinea-pig and pigeon, the protoplasm has become increased, and contains a considerable number of globules of myelin. A new phenomenon now commences, the multiplication of the interannular nuclei. It begins in the nerves of the animals just mentioned toward the fifth day. Now, in the proper preparation, we are able to distinguish five or more successive stages of this phenomenon. In the first we recognize that the nucleus is simply enlarged and contains a very large nucleolus. In the second the nucleus is still larger, and the nucleolus is in the form of a purse (*bissac*). In the third stage the nucleus is more voluminous, and contains two nucleoli, more or less separated. In the fourth the nucleus itself, which encloses two nucleoli, takes the form of a purse. Sometimes, instead of possessing a depression on each side like an old-fashioned purse, there may be a slight depression on one side, or the same may be deeper, as if cut with a sharp instrument. In the fifth stage two nuclei are seen near each other, each possessing a nucleolus. The nuclei are smaller than those seen in the other stages. It is evident that the original nucleolus first becomes divided, then the nucleus, at first presenting a depression at its centre, finally separates into two nuclei of new formation. In a more advanced stage these new nuclei have each divided, and we can see four nuclei, each with a nucleolus, side by side, slightly separated or not by protoplasm. Each nucleus always preserves a rounded form in this degeneration. That there is really a multiplication of nuclei instead of bringing to view normally-existing nuclei (obscured by the myelin) by the resorption of the myelin, as Schiff held, may be demonstrated by an hourly, as it were, examination, for two or three days, of a frog's pneumogastric nerve, which had been divided twenty-five to thirty days previous (corresponding to the fifth day of degeneration in the nerves of the rabbit or rat).

*Course from the Fourth to the Twenty-fifth Day.*—After the fourth day the increase of protoplasm, multiplication of nuclei, and segmentation of the myelin into smaller globules continue. At a later period, which cannot be precisely determined, the finely-segmentated myelin accumulates at certain points of the nerve-tube, and the nuclei multiply. The tube is enlarged at the points of accumulated myelin; elsewhere it is turned upon itself and its calibre is filled by elongated nuclei, arranged in series, and by the protoplasm, which, from the eleventh to the twentieth day, shows longitudinal striæ, due to a special arrangement of this protoplasm within the sheath of Schwann. The nuclei are separated from each other by a smaller or larger quantity of myelinic globules. This separation of the nuclei and the interposition of the myelin-globules is probably due to ameboid movements of the protoplasm.

*From the fifteenth to the twenty-fifth day*, rounded or fusiform masses of myelin, with two *fringed* ends, may be seen.

*From the twentieth to the seventy-second day*, the phenomena just described progress very slowly. During this period the nerve-tubes are not larger than normal; some are even more slender.

As in the interannular protoplasm, so, too, there is an infiltration of fat-granules into the protoplasm of the connective-tissue cells of the perifascicular sheath, of the endothelial cells of the laminated sheath, and blood vessels of the nerve.

Evidently this so-called degeneration is improperly named. Instead of being a passive or degenerative process, it is *active.* No one would think of calling osteitis a passive phenomenon. Nevertheless the histological sequences are the same in this so-called degeneration of nerves as in osteitis. The increase of protoplasm, swelling of the interannular nucleus and its multiplication, and the subsequent division of the axis-cylinder at various points within the interannular segment, is a sequence of events which shows the process to be one of activity. The axis-cylinder is divided and absorbed as a consequence of the preceding events; it is not itself a foreign body, as it were, developing activity.

*Changes in the Central and Peripheral Bulbs.*—Heretofore we have been limited to the examination of a segment exsected from the peripheral portion of the divided nerve at a short distance posterior to the *peripheral bulb* (*bourgeon*), as we may designate the ultimate proximal peripheral end for convenience's sake. We may also call the immediate extremity of the central end, *the central* bulb. The morphological changes which occur in these bulbs are as follows:

The division of a nerve gives rise to a slight effusion of blood and myelin. Vascular hæmostasis, and slight inflammation, with oozing of leucocytes about the ends of the nerve, immediately follow. Then there is swelling of the tubes, due to absorption of the surrounding plasma; the latter distends Schwann's sheath and expels the myelin, which becomes diffused between the nerve-tubes. All this occurs on the first or second day after section.

At the beginning of the fourth day the central and peripheral ends are slightly swollen, and rose-colored, due to the effusion of red blood-corpuscles and congestion. The enlargements at these ends form the *terminal bulbs*, and are caused by a swelling of the myelin, and the presence of a large quantity of lymphatic cells charged with globules of myelin and red blood-corpuscles.

Between the third and twelfth days a special preparation of these bulbs would enable us to see:

*In the Central Bulb.*—The axis-cylinder, in the majority of the nerve-tubes, so hypertrophied as to completely occupy, for a considerable length of the tube, the whole calibre of Schwann's sheath.

The longitudinal striation of the axis-cylinder more distinct than normal.

At certain points, groups of fatty granules or globules of myelin between the axis-cylinder and Schwann's sheath.

At certain other points complete preservation of the myelin. In tinted preparations, elongated and flattened nuclei, enclosed within a group of small fat-granules. These granular groups correspond to lymphatic cells, since the latter are found in abundance about the extremities of the divided nerve, and, having absorbed a quantity of fatty granules and myelin-globules, they insinuate themselves between Schwann's sheath and the axis-cylinder, penetrating the nerve-tubes to a greater or lesser distance. Arrived here, they gradually absorb the surrounding myelin. Red blood-globules may also be found within the nerve-tubes. They reach the interior of the latter by passively flowing, after having escaped from the vessels divided on section of nerve, with the plasma to the space between Schwann's sheath and the axis-cylinder, now left gaping since the myelin has escaped, and they attain a certain height within the tubes. The progress of the development of these phenomena is irregular, by no means uniform in each tube. Finally, the lymphatic cells which have wandered within the nerve-tubes of the central bulb, after having attacked the isolated globules of myelin, then the myelinic layer, subsequently attack the axis-cylinder itself. The latter disappears in the immediate vicinity of the divided end, but *a little above this it is constantly found.* These changes in the nerve-tubes of the central bulb extend in some as far as the first annular constriction, in others not as far. In the latter case a portion of the interannular segment, above the point of section, is preserved in its normal condition. In certain cases, again, these alterations may even extend beyond the first annular constriction; and these changes consist in that the interannular segment, situated above the first constriction, instead of containing a single nucleus, possesses several, which are imbedded in common protoplasmic layer, disposed between the myelin and Schwann's sheath.

*In the Peripheral Bulb.*—A similar investigation of the nerve-tubes of the peripheral bulb demonstrates changes analogous to those described, with this difference, *the axes-cylinder disappear.*

Toward the twelfth day, the myelin, having become granular, has escaped from the patulous Schwann's sheath for quite a distance, so that the degenerated nerve-tubes only here and there contain globules of myelin, and are almost uniformly constituted by their vacant Schwann's sheath, which is turned back upon itself.

Such is the condition in which the different parts of the nerve-tube are found when regeneration commences. These changes invade the sensory as well as the motor nerves.

*Regeneration of Nerves.*—The process of nerve regeneration is begun, in the rabbit, about the twentieth day. As we have already seen that the axes-cylinder remain intact in the nerve-tubes of the central bulbs, the process of regeneration essentially consists in the prolongation of these axes-cylinder from the central to the peripheral bulbs, thence between or within the sheaths of Schwann, remaining in the peripheral nerve segment, to its ultimate extremities. Eventually these axes-cylinder acquire their medullary sheaths, becoming perfect myelinic nerve-tubes of new formation.

Special examination of the central and peripheral bulbs, the peripheral segment of the nerve, and the cicatricial segment, would demonstrate these respective stages.

*Central Bulb.*— Proper preparations show the following between the twentieth and the one hundred and sixtieth days:

1. From a primitive nerve-tube, with a slightly swollen extremity, there shoots forward a tube with a delicate sheath of myelin, characterized, nevertheless, by annular constrictions and interannular nuclei. Between this newly-formed tube and the old Schwann's sheath which covers it, a granular mass with large nuclei is distinguished.

2. From the terminal bulb of a normal nerve-tube an axis-cylinder becomes disengaged; at a short distance it divides into two or more parts, each of which becomes a myelinic nerve-tube.

3. From the extremity of a primitive nerve-tube several delicate, but perfect, myelinic nerve-tubes spring; in their onward course they entwine one another.

4. From an old nerve-tube a smaller myelinic nerve-tube departs, around which a still more delicate nerve-tube is entwined; the latter arises at a higher point than the original tube.

5. From an original nerve-tube bulb a more delicate myelinic nerve-tube is continued a certain distance, where it acquires abruptly the size of the parent tube, all being contained within an unbroken sheath of Schwann; the more delicate part is really an interannular segment, recognized by its central nucleus.

6. This is the commonest disposition: From the extremity of an original myelinic nerve-tube a slender tube shoots forward a certain distance, then divides into two tubes of almost equal size with itself; after a short course, each of the latter divides; this division continues, so we may further on see a single Schwann's sheath enclose eight or more tubes. Schwann's sheath is recognized by the globules of myelin found at irregular distances.*

*Cicatricial Segment.*—This is seen to be composed of a very considerable number of small nerve fasciculi. They contain neither myelinic nor fatty globules. They are enveloped by a very delicate sheath, carpeted on its under surface by endothelial cells; it is, in fact, analogous to Henle's sheath. These fasciculi contain both myelinic and non-myelinic tubes, their relative number varying. Certain fasciculi contain non-myelinic tubes exclusively. These differences may be accounted for by the *kind* of nerve, and *time* of observation.

*Regarding the formation of nerve-cells (bi- or poly-polar) within the central bulb, which S. Mayer (*Archiv. f. Psychiatrie*, 1876, p. 430) claims to take place, I will say that in the pneumogastric nerve, sixty days after section, I have observed, in the central bulb, cicatricial segment, and peripheral bulb, refracting homogeneous globes, more or less deeply tinted gray by the osmic acid, usually bi-polar, and seemingly placed over a non-myelinic nerve-tube. But, inasmuch as I have never been able to detect a nucleus within them, they should not be considered as nerve-cells.

The course of the tubes is not straight in all, some taking an oblique or almost perpendicular direction, others intercrossing in an irregular manner.

*Peripheral Segment.*—If examined at the end of sixty or seventy days following section of a pneumogastric nerve, fixed with osmic acid, and disassociated with care, we should see a large number of pale nerve-tubes, each furnished with nuclei here and there. Other fibres, presenting globules of myelin within their sheaths, are also seen. Both arise from myelinic tubes, possess nuclei, and present a longitudinal striation. Besides these, a third species exists, possessing globules of myelin here and there, and within their interior a regenerated nerve-tube is to be distinguished. The latter is very delicate and presents a bluish-gray appearance, due to osmic acid staining of its myelin. It possesses perfectly clear annular constrictions, but they are nearer each other than normally. Each of the latter has its central nucleus. In one of these small tubes, the diameter of which is $\frac{4}{1000}$ MM, the annular constrictions are ranged at a distance of $\frac{150}{1000}$ MM. In some original tubes, two, four or more newly-formed tubes may be seen. A fourth species is found, namely: a nerve-tube, either free or contained within an original Schwann's sheath, which at a certain point loses its myelin, is continued as a non-myelinic tube, then abruptly retakes its myelinic sheath. Such are the principal types met with in the peripheral segment.

From the hundredth to the hundred and sixtieth day (even at the sixtieth day, sometimes) we may see myelinic nerve-tubes of new formation within the peripheral segment, which are perfectly free, not enveloped by old sheaths of Schwann. This species is often accompanied by a turning non-myelinic tube.

*Mode of Union of Both Ends.*—The delicate fasciculi of the cicatricial segment are prolonged in full calibre from the original nerve-tubes of the central bulb. Each nerve-tube of the cicatricial segment is surrounded, not by Schwann's sheath, but is bathed in a plasma in which various cells float. It is probable that this plasma-sheath is formed at the expense of lymphatic and connective cells of the cicatricial tissue.

The point of soldering of the cicatricial segment and peripheral bulb can be distinguished by the sheaths of Schwann, containing ovoid masses of myelin and fatty granules. Besides, the degenerated nerve-tubes of the peripheral segment present very delicate fatty granules throughout their whole length.

Within these old sheaths of Schwann the newly-formed nerve-tubes are seen to be prolonged from the cicatricial tubes. Thus comes the conclusion that the newly-formed nerve-tubes seen within the peripheral bulb and segment take their origin from the central segment.

